# The related factors of compliance to professional codes of ethics from midwives' perspective working in healthcare centers of Tehran-Iran

**DOI:** 10.11604/pamj.2018.30.40.14436

**Published:** 2018-05-17

**Authors:** Leila Nasiriani, Seyedeh Fatemeh Vasegh Rahimparvar, Tahmineh Farajkhoda, Naser Bahrani

**Affiliations:** 1 School of Nursing and Midwifery, Tehran University of Medical Sciences, Tehran, Iran; 2 Nursing and Midwifery Care Research Center, School of Nursing and Midwifery, Tehran University of Medical Sciences, Tehran, Iran; 3 Research Center for Nursing and Midwifery Care, Shahid Sadoghi University of Medical Sciences, Yazd, Iran

**Keywords:** Midwifery, codes of ethics, Healthcare Centers (HCCs), Iran

## Abstract

**Introduction:**

Professional ethics is an important issue in every organization. This study was designed to define compliance level to professional codes of ethics and some of its related factors in midwives working at healthcare centers.

**Methods:**

This cross sectional study was conducted in 2013 Tehran Iran. 125 midwives from the governmental healthcare centers of Tehran were selected through stratified quota sampling method. Data gathering tool was a self-reporting questionnaire which included: demographic characteristics, Iranian version of ethical codes of reproductive health providers, applicability of these ethical codes and awareness about the professional codes of ethical. Data analysis was conducted using SPSS v.16. p level at 0.05.

**Results:**

Compliance to the professional ethical codes were directly correlated to the income level of the midwives, the applicability of the codes and the midwives' awareness about them.

**Conclusion:**

It is necessary to pay attention to professional ethics and its teaching as such. It is also important to monitor compliance to the ethical codes. Moreover, living conditions of the midwives should be one of the priorities to enhance their performance.

## Introduction

Medical ethics is an interdisciplinary subject and its content is moral values of medicine. Therefore, the necessity of compliance to the moral values and the uniformity in care and the treatment behavior are of critical importance [1]. Experts in medical ethics emphasize on the widespread use of ethical codes in the field of health care [2-4]. Ethical performance is one of the main factors in providing quality care to women and mothers. It is necessary for the health care personnel, at any position of service, to base their performance on common values which are indicators of their loyalty to the society and their profession as well [5]. All over the world, midwives satisfaction is one of the main requirements for maintaining and improving reproductive health [6, 7]. Midwives are responsible for the safety of women and supporting their empowerment. Therefore, they would encounter many important ethical issues during their professional activities [8, 9]. Paying attention to all ages is one of the characteristics of the ethical codes of the health care providers [10]. Compliance to the principles and the ethical codes of midwifery would result in providing fair treatment/care, protecting patients/clients from unnecessary risks, enhancing recovery, improving and increasing patient's pain tolerance level, reducing patient's anxiety, making motivation for the patient to have a better cooperation with the medical team, reducing treatment costs, increasing satisfaction and strengthening mutual trust between the therapist and the patient [11]. It has been continuously suggested in recent years to consider medical ethics at three levels: 1) educating the professional ethics to the students, 2) compliance to the ethics during care or treatment and 3) managerial and executive level for the public health [12]. The experts of every profession should evaluate the level of compliance to the professional codes of ethics in their profession. In other words, to improve the performance of the system, experts need to study the ethical codes, assess the level of compliance and investigate factors that would affect them [13]. Reviewing relevant scientific sources revealed that there were not enough studies on how to check compliance to the ethical codes in providing midwifery care and treatment in Iran. Therefore, to assess the ethical performance of midwives, this study was designed to evaluate the compliance to the codes of ethics and its related factors among midwives in Tehran's governmental HCCs. It is hoped that by knowing the performance of the midwives regarding the compliance to the ethical codes and also by knowing the factors affecting this compliance, which is a fundamental pillar of the health care system, a step, as small as it may, would be taken towards more effective programming for the compliance to the ethical principle.

## Methods

This is a cross-sectional study that was conducted in Tehran-Iran in 2013. The sample size was calculated to be 125 midwives. The inclusion criteria were: having midwifery and having at least six months of work experience in the field of midwifery. The exclusion criteria were participant´s willingness to withdraw from the study (in fact, nobody withdrew from the study). Sampling was conducted using stratified quota method. According to this method, Tehran had six governmental HCCs, and each one was considered as one stratum. Each one of them had several subsets: Northern (with 22 subsets), Southern (with 31 subsets), Eastern (with 22 subsets), Western (with 16 subsets), Shemiranat (with 14 subsets) and North Western (with 13 subsets). The number of midwives who were covered by each category was determined. Then, the quota was determined in accordance with that category. However, considering the possibility of sample loss, 6 to 8 more samples were selected from each primary governmental HCCs. Thus, a total number of 162 midwives accidentally received questionnaires. The data that were collected through self-reporting questionnaires included: Demographic characteristics, Codes of ethics questionnaire for reproductive health providers (RHP) in two sections consisting of compliance to the codes (health care providers' codes of ethics) and the applicability of the codes and the awareness of about 10 ethical points in midwifery. The RHP codes of ethics were Iranian version of questionnaires that FarajKhoda et al (2012) evaluated and designed through an exploratory and mixed study of all reproductive health providers like midwives. Its psychometric indicated 0.94 content validity index, 94% consensus of the panel of experts, internal consistency of 86% and stability of 95% [8].

The questionnaire was named the RHP questionnaire. The questionnaire contained 19 main domains. However, in this study, only 10 domains of the questionnaires were examined. These domains included: respect for patient's dignity (4 questions), patient's right for making decisions (8 questions), taking informed consent (2 questions), presenting medical information to the patients (4 questions), confidentiality of the patient's information (5 questions), disclosing the patient's information to the affected community (4 questions), respecting the patient's privacy (5 questions), benefiting and not harming the patient (6 questions), having justice (2 questions) and having professional relationship with colleagues (3 questions). In this study, the questionnaire contained 43 questions. Five-point Likert scale was used to measure the compliance to these codes in a way that 1 indicated very low, 2 was low, 3 was average, 4 indicated high and 5 meant very high. Therefore, the minimum score of the questionnaire was 43 and its maximum score was 215. This questionnaire was used in two parts: to assess the compliance to RHP codes of ethics, and to review the applicability of these codes. Since the number of questions in various levels were different, to compare the values of different levels, the value of each level and the total value of the compliance level/usage were reported in percentage. Method to report the results was: lower than 25% compliance meant low, 25%-50% compliance meant average, 50%-75% compliance meant fair or higher than average and 75%-100% compliance meant good. About 10 ethical points in the awareness questionnaire of midwifery was researcher-made. This questionnaire contained 10 multiple-choice questions. Score 1 was given to the right answer, and score 0 was given to the wrong answer. The minimum score for this questionnaire was 0 and its maximum score was 10. The validity of this questionnaire was evaluated through content validity and CVI and was 0.89. The correlation coefficient of 0.94 was found by test-retest method as good reliability. Participants completed the questionnaire after explaining the aims of the research and convincing the midwives to have integrity and honesty. SPSS software version 16 was used to describe and analyze the collected data. Pearson and Spearman correlation coefficient were used to determine the correlation between the variables [table 4]. A significant level was set at p<0.05. This study was supported by Tehran University of Medical Sciences and the ethical committee of this university approved the study (250/1682. Date: 2012/4/15).

## Results

125 questionnaires were completed without defects. The demographic characteristics are presented in Table 1. All the data were normal, according to Kolmogorov-Smirnov test. The minimum score that was obtained for the compliance to the HCP ethical codes from the participants of this study was 109, and their maximum score was 207. The mean of the compliance to the HCP ethical codes from the viewpoint of midwives was in good level of compliance: 70.93 ± 12.42 . The levels of compliance to each 10 domains are shown in Table 2. The applicability of each 10 domains are shown in Table 2. In this study, the mean of the awareness about the ethical points in midwifery was 4.6 ± 1.67. The rest of the specifications are listed in Table 3. Using Spearman and pearson correlation coefficient, it was identified that the compliance to the HCP ethical codes has a significant association with the midwives' income (p = 0.004). This was defined in a way that the more desirable income from the participants' point of view, the more compliance they showed to the HCP ethical codes. It was also identified that the compliance to the HCP ethical codes had a significant association with the applicability of these codes and with the awareness of the ethical points in midwifery as well (p=0.001) (Table 3).

**Table 1:** Demographic characteristics of midwifes working in Tehran’s governmental HCCs in 2013

Demographic characteristics	
Age (year) (mean ±SD)	37.38± 8.34
Time passed since graduation	12.64± 8.31
Duration of working at the current health center	9.3 ± 6.13
**Marital status n (%)**	
Single	30 (24.0%)
Married	95 (76.0%)
**Income status n (%)**	
Desirable	11 (8.8 %)
Average	66 (52.8%)
Undesirable	48 (38.4%)
**Attending educational classes about the professional ethics (optional)**	
Never attended	74 (59.2%)
workshop	30 (%)
Attending seminars and congresses	19 (15.2)
Education during employment	28 (22.4 %)
**Method of acquiring information about the professional ethics**	
Don’t have any information	24 (19.2%)
Personal study	30 (24%)
Educational courses at university	58 (46.4 %)
Education during employment	31 (24.8%)
Workshop	19 (15.2%)
**Employment status**	
Official	74 (59%)
Project base	19 (15.2%)
Semi official	6 (4.8%)
Contract base	26 (20.8%)
Educational level	
Associate degree	15 (12%)
Bachelor’s degree	100 (80)
Master’s degree	10 (8)

**Table 2:** Minimum, maximum and mean scores of compliances to and applicability of the HCP ethical codes by midwifes working in Tehran’s governmental HCCs 2013

Categories of the codes	Compliance		applicability	
	Min-Max	Mean ±SD	Min-Max	Mean ± SD
Patient’s human dignity right	31.25-100	74.37 ±8.12	18.75-100	66.56±8.62
Patient’s right for decision making	21.87-90.65	65.25 ±10.41	0-93.75	58.83±10.56
Acquiring informed consent from patient	0-100	62.12 ±6.57	0-100	57.62±6.37
Revealing information to the patient	0-100	75 ±8.12	0-100	68.62±7.54
Patient’s right for confidentiality of their information	20-100	70.91 ±7.26	0-100	67.7± 7.2
Revealing patient’s information	12.5-100	61.94 ±11.34	0-100	59.12 ±8.34
Preserving patient’s privacy	25-100	70.35±9.36	25-100	73.5 ±8.57
Benefiting and not harming the patient	33.33-100	74.17 ±9.16	12.5-100	82.96±11.39
Justice toward the patient	0-100	77.5 ±6.48	0-100	72.5±12.84
Professional relationship with colleague	25-100	80.83±6.67	16.67-100	75.83±14.83
Total	38.37-95.35	70.93 ±12.42	18.86 -95.93	156±26.150

**Table 3:** Compliance to the HCP ethical codes based on some variables in Tehran’s governmental HCCs’ midwives in 2013

variables	Compliance to the HCP ethical codes	
	correlation coefficient	P value
Age	r = 0.071^*^	0.2
Employment status	r_s_= 0.08	0.3
Educational level	r_s_ = 0.12	0.3
Time passed since graduation	r_s_ =0.065	0.2
Duration of working at the current health center	r_s_ = 0.091	0.3
Marital status	r_s_ = 075	0.8
Income status	r_s_ = 0.11	0.04
Attending educational classes about the professional ethics	r_s_ = 0.068	0.3
Method of acquiring information about the professional ethics	r_s_ = 0.057	0.2
The level of applicability of codes	r =0.32^*^	0.001
The level of awareness about the codes	r =0.29^*^	0.001

r= Pearson correlation coefficient (for Numerical variable), r_s_ = Spearman correlation coefficient (for categorical variables)

## Discussion

This study was designed to assess the level of compliance to the HCP ethical codes and the related factors from midwives' point of view working in governmental HCCs in Tehran-Iran. Since no similar study was found about the HCCs, the environment for this research was chosen to be hospitals and the HCCs as well. This could be the main reason for having different results. Compliance to the ethical principles guarantees the rights of the patients [8]. Therefore, many studies were conducted on respecting the rights of the patients in Iranian hospitals. However, there are very little studies about the performance of the HCCs' staff towards the patients in Iran. In this study, the compliance to the HCP ethical codes by midwives in governmental HCCs of Tehran was at a good level. According to Vasheghani et al, compliance to the nursing codes of ethics was 63% [14]. Rad et al reported that compliance to patients' rights by medical staff was poor in 75% of the cases, very poor in 20% and average in 5% cases [15]. Baghani et al also stated that the level of compliance to the ethical principles in the field of professional midwifery responsibility and midwifery relationship were 50% and 86%, respectively. In addition, compliance was higher among the 7^th^ semester students compared to the 5^th^ semester students [16]. In Bazrafkan's study, compliance to the ethical codes by medical staff was at an appropriate level of 63% [1], which is in line with the results of the present research. Khodakarami et al found that 87% of the pregnant mothers never received any information about the type of the childbirth, the advantages/disadvantages of different childbirth methods, their rights during pregnancy and childbirth after delivery [6]. According to Faraj Khoda et al, more attention should be paid to the rights of the patients, especially women [5]. In this study, the level of compliance to human dignity of the patients, from the perspective of the midwives in HCCs of Tehran, was 74.37 ±8.12. It was found, in a study performed by Manookian et al, that the factors affecting respect for human dignity of the patients consisted of the personality of the nurse (such as personal beliefs, behavior, communication, spending enough time in the field, the role of nursing staff, having professional commitment, the number of personnel and the competence of the staff). Results showed that to have a better performance in respecting human dignity of the patients, it is necessary to increase the nurses' knowledge about human dignity and the factors affecting it [17]. Also, another study showed that human dignity plays an important role in patients' care according to the health care staff or the patients [18-20].

In the present study, the level of compliance to the patients' rights of making decision, by midwives was at an appropriate level of 65.25 ± 10.4. In this regard, Motevalizadeh et al reported that the level of respecting the patients' independence, who referred to the public hospitals around Tehran, was 64.7% [19]. As well, several studies showed the importance of patients'/clients' decision-making from the viewpoint of the health providers [10, 21-23]. Mendick et al. noted that the compliance to the decision making right from the perspective of physicians or the HCP and the patients/clients' have many variations. And many factors affect the compliance to this right. As such, improving the responsibility of the patients/clients in their health care decisions would reduce the responsibility of the medical team [18]. Midwives who participated in the present study had a 62.12 ± 6.57 rate of compliance in taking informed consent from the patients, which was considered as a fair level. According to the general law in Iran, patient's treatment without their consent is considered battery and assault. However, if the informed contest is incomplete or not taken correctly, it would be considered as a misconduct and neglect in performing the duty [24]. Briks et al found the importance of taking informed consent from the patients [25]. Studies also suggested the improvement of the providers' knowledge about taking consent form, since they did not have an appropriate compliance level in the past [24,25]. In the present study, the level of compliance to information revelation (truthfulness) to the patients by midwives in the HCCs was 75 ± 8.12 In modern medical ethics, the therapist must provide the necessary information and the available factors as well, to the patient so that the patient could make informed decision about the diagnostic steps and the treatment practices. It seems that understatements are common in Eastern countries [2]. A study by Zamani et al revealed that 88% of the patients and 90% of the physicians agreed to disclose the information and tell the truth to the cancer patients at early stages of their disease. Also, 78% of the patients and 72% of the physicians agreed to tell the truth about the disease to the patient with advanced cancer as well [20]. Additionally, several studies showed the importance of truthfulness. In most cases, enough information about the cost and the insurance services is not given to the patients [21]. In this study, the mean level of compliance to the right of maintaining client's confidential information by midwives was 70.91 ± 7.26 ,which was at a satisfactory level. It should be noted that confidentiality is one of the eldest rules of medical profession. It would benefit the personal interests of the patient, the physician and the public [23]. A study conducted by Lemonidou et al. about professional secrecy concluded that patients believed their secrets were not preserved, while nurses believed the opposite is occurring [22]. In fact, it seems that the health provider's opinion is different from the patient's in terms of confidentiality and other ethical domains.

In the current study, the mean of compliance to the patients' information disclosure law by midwives was 61.94 ± 11.34 ,which indicates that the compliance to the disclosure of clients' information is at a fair level. Yarmohammadian et al. reported that in Iran, like all other five countries that were examined in their study, there is a written policy for disclosure of the health information to judicial authorities. The policy states that the health centers and their staff, with patient's permission, should only disclose patient's medical records to the court, the representatives of judicial authorities and the law enforcement agencies, unless the court has given a judicial warranty disclosure [24]. Numerous obstacles have been expressed for the disclosure of information by the medical team. The knowledge of the medical professionals about the disclosure policies to the clients is one of the success factors in the implementation of this ethical principle [25]. In the present study, the mean of compliance to patient's privacy was 70.35 ± 9.36 .Based on this, the level of compliance was good. Lemonidou et al found that the mean of compliance to the privacy by the nurses was 3.05 ± 0.80, while their patients believed that (on average) this amount was 2.36 ± 0.99 [22]. It seems that the necessary conditions for the compliance to patient's privacy are available more at the health centers compared with the hospitals. In terms of respecting the privacy of the patients and keeping their information confidential, the compliance to patients' rights is not at a desirable level for the hospitalized patients [21]. In the present study, the mean level of compliance to the ethical codes to benefit the patients and not harming them by midwives, was 74.17 ± 9.16, which is at a good level. In terms of the ethical considerations for surrogacy, the principle of not harming the child and the surrogate mother includes screening the biological parents to check for infection with hepatitis C, B and HIV. This compliance is necessary for the protection of the surrogate mother [26]. In the present study, the level of fairness towards the client/patients by the midwives was 77.5 ± 6.48, which is at a good level. In a study by Khademolhosseini et al, it was found that the compliance to fairness by the physicians is the most important factor, from both the patients' and the physicians' point of view, in decreasing disobedience from the physicians' orders. Also, among the indicators related to the justice, from the physicians' point of view, “Choosing the best and the most effective treatment,” and from the patients' view “physicians” sense of responsibility” had the highest score [27]. As well, in the present study, the high scores obtained from the midwives may not be the same in the patients' opinion. In the current study, the mean of compliance to the professional relationship with colleagues by the midwives was 80.83 ± 6.67, which is at a good level of compliance. Appropriate and effective relationship between members of the medical team leads to a better compliance to the principles of professional ethics and results in higher patients' satisfaction. It seems that professional stress in the workplace (hospitals and the HCCs) had an important effect on the satisfaction with the colleagues. Psaila et al showed that the success of the health care system is directly related to good behavior and correct interactions between the professionals working together [28].

In this paper, it was tried to work on some factors related to the compliance to the HCP ethical codes from the perspective of the midwives working at Tehran's governmental HCCs. Several different factors would influence the compliance to the ethical principles [29]. Mendick et al. claimed that the compliance to the ethical principles is hugely different between the health care providers' and the patients/clients' point of view. They also stated that different issues would affect their compliance [18]. Analysis of the data from the present study determined that compliance to the HCP ethical codes had no statistically meaningful relationship with age, work experience, employment status and marital status. There are also studies in the literature showing the same results [1,30,31]. However, in a study conducted by Rafii et al., there was a meaningful relation between age and compliance to the professional ethical codes [32]. In this study, there was no statistically meaningful relation between the level of education and compliance to the HCP ethical codes. The study of Ajami et al also determined that there is no meaningful relation between the rate of compliance to the patients' rights, the employment status and the educational level [30]. Mahmoudian et al suggested no meaningful relation was found between age and gender with the compliance to the professional ethical codes. However, there was a meaningful relation between the educational level and the compliance to the professional ethical codes [33]. In the present study, compliance to the HCP ethical codes had a positive correlation with the income level in a way that the higher the income level, the higher the level of compliance to the HCP ethical codes. Also, Dehghani et al showed the same relationship [31]. In the present study, compliance to the HCP ethical codes had a direct statistical correlation with the level of awareness about the ethical points in midwifery. In fact, by increasing the awareness, the compliance to the HCP ethical codes increases as well. Feisal et al believed it is desirable to increase midwives' awareness about the medical ethics and the professional principles as such [34]. Faraj Khoda et al lso claimed that the lack of awareness among the medical service team would result in violation of the professional ethical principles [35]. Borhani et al concluded that teaching the ethical principles and assessing their compliance would increase students', as well as service providers', sensitivity to this issue. Consequently, compliance to the ethical codes would increase as well [36]. Sheikh Taheri et al concluded that although the nurses' awareness about the patients' rights was at an acceptable level, the compliance to the patients' rights was not satisfactory [37]. In the present study, compliance to the HCP ethical codes had a statistically meaningful relationship with their applicability. Khodaparast et al claimed in their study that the ethical codes could only be applicable when the design is based on the needs of the professionals and also based on the professional ethical/legal duties [38]. This result is similar to the present study and is indicative of the practicality of the codes.

## Conclusion

This study showed that the level of compliance to the HCP ethical codes by midwives of HCCs in Tehran-Iran was higher than average. It was also shown that the income level, the applicability of the HCP ethical codes and the awareness of the midwifery ethical pointes have a direct statistical correlation with the compliance to the codes by the midwifes. It is suggested that to design the ethical codes, their applicability should be taken into consideration.

### What is known about this topic

- Ethical performance is one of the main factors in providing quality care to women and mothers;
- Compliance to the ethical principles guarantees the rights of the patients;
- By Complying to the professional ethical principles, clients/patients and the colleagues trust midwives.

### What this study adds

- Level of compliance to the HCP ethical codes by midwives of HCCs in Tehran-Iran was higher than average;
- Income level, the applicability of the HCP ethical codes and the awareness about the ethical pointes had a direct statistical correlation with the compliance to the codes by the midwifes.

## Competing interests

The authors declare no competing interests.

## References

[1] Bazrafcan L, Nabeiei P, Shokrpour N, Moadab N (2015). Medical ethics as practiced by students, nurses and faculty members in Shiraz University of Medical Sciences. J Adv Med Educ Prof.

[2] Foster IR, Lasser J (2010). Professional ethics in midwifery practice.

[3] Jonsen AR, Siegler M, Winslade WJ (1982). Clinical ethics a practical approach to ethical decisions in clinical medicine.

[4] Oshima Lee E, Emanuel EJ (2013). Shared decision making to improve care and reduce costs. N Engl J Med.

[5] Farajkhoda T (2012). Developing the professional codes of ethics for reproductive health care provider and it's assessment from their viewpoints in Yazd Health care centers.

[6] Khodakarami N, Jan Nesari S (2009). Evaluating mothers' awareness about pregnant women's rights. J Med Ethics Hist Med.

[7] Kiani M, Ahmadi M, Azimi N, Majd HA (2016). A survey on observing patient's autonomy in the labour sections of chosen hospitals of Tehran by students of medical science department. Medical Ethics Journal.

[8] Farajkhoda T, Roudsari RL, Abbasi M (2013). An exploratory study to develop a practical ethical framework for reproductive health research. Iran J Reprod Med.

[9] Rahimparvar V, Nasiriani L, Faraj Khoda T, Bahrani N (2014). Compliance rate of midwives with the professional codes of ethics in Maternal Child Health Centers in Tehran. J Med Ethics Hist Med.

[10] Kuther TL (2003). Medical decision-making and minors: issues of consent and assent. Adolescence.

[11] Hammami MM, Al-Jawarneh Y, Hammami MB, Al Qadire M (2014). Information disclosure in clinical informed consent: “reasonable” patient's perception of norm in high-context communication culture. BMC Med Ethics.

[12] Del Pozo PR, Fins JJ (2005). The globalization of education in medical ethics and humanities: Evolving pedagogy at Weill Cornell Medical College in Qatar. Acad Med.

[13] Limentani AE (1999). The role of ethical principles in health care and the implications for ethical codes. J Med Ethics.

[14] Vasheghani Farahani A, Maleki M, Golpira R, Bakhshandeh H, Parsa M, Mayel Afshar M (2015). Perspectives of doctors, nurses and patients on physicians' observance of professional ethics. J Med Ethics Hist Med.

[15] Rad M, Mohammd A, Esna Ashari P (2004). Patients and physicians awareness of patients' rights and its implementation at Beheshti hospital in Isfahan. Iranian Journal of Medical Education.

[16] Baghany R, Faramarzi F, Baghjari M, Zare F, Tabaraei Y (2013). Observance of Midwives' Code of Ethics by Midwifery students during vaginal examinations in labor and its influencing factors in 2012. Journal of Mazandaran University of Medical Sciences.

[17] Manookian A, Cheraghi MA, Nasrabadi AN (2014). Factors influencing patients' dignity: a qualitative study. Nurs ethics.

[18] Mendick N, Young B, Holcombe C, Salmon P (2010). The ethics of responsibility and ownership in decision-making about treatment for breast cancer: triangulation of consultation with patient and surgeon perspectives. Soc Sci Med.

[19] Motevallizadeh S, Afzali HM, Larijani B (2011). Observing principles of medical ethics during family planning services at Tehran urban healthcare centers in 2007. Iran J Reprod Med.

[20] Zamani A, Shahsanai A, Kivan S, Hematti S, Mokarian F (2011). Iranian Physicians and Patients Attitude toward Truth Telling of Cancer. Journal of Isfahan Medical School.

[21] Anbari Z, Mohammadi M, Taheri M (2015). Satisfying patients' rights in Iran: Providing effective strategies. Iran J Nurs Midwifery Res.

[22] Lemonidou C, Merkouris A, Leino-Kilpi H, Välimäki M, Dassen T, Gasull M (2003). A comparison of surgical patients' and nurses' perceptions of patients' autonomy, privacy and informed consent in nursing interventions. Clin Eff Nurs.

[23] Nazari Tavakoli S, Nejadsarvari N (2013). Confidentiality: a comparative study between medical ethics principles and Islamic ethics. J Med Ethics Hist Med.

[24] Yarmohammadian MH, Raeisi AR, Tavakoli N, Nansa LG (2010). Medical record information disclosure laws and policies among selected countries; a comparative study. J Res Med Sci.

[25] Birks YF, Harrison R, Bosanquet K, Hall J, Harden M, Entwistle V (2014). An exploration of the implementation of open disclosure of adverse events in the UK: a scoping review and qualitative exploration.

[26] Asghari F (2008). Ethical issues in surrogate motherhood. J Reprod Infertil.

[27] Khademalhosseini Z, Khademolhosseini M, Mahmoodian F (2009). A study on the ethical and behavioral role of physician in following the medical treatment plan by the patient during the treatment. J Med Ethics.

[28] Psaila K, Schmied V, Fowler C, Kruske S (2014). Discontinuities between maternity and child and family health services: health professional's perceptions. BMC Health Serv Res.

[29] Choe SY, Min K-H (2011). Who makes utilitarian judgments? The influences of emotions on utilitarian judgments. Judgm Decis Mak.

[30] Ajami GRV, Foroughan M, Hosseini M (2012). The Relationship between Knowledge and Observance of Patients' rights in Rehabilitation Centers of Tehran. Journal Of Sabzevar University Of Medical Sciences.

[31] Dehghani A, Mosalanejad L, Dehghan-Nayeri N (2015). Factors affecting professional ethics in nursing practice in Iran: a qualitative study. BMC med ethics.

[32] Rafieei M, Mohammadi N, Shobeiri F, Roshanaei G (2014). Awareness rate of nurses & midwives working in the hospitals of Hamadan on principals of professional ethics in 2013. Pajouhan Scientific Journal.

[33] Mahmoudian F, Tabei SZ, Nabeiei P, Moadab N, Mardani M, Sarvestani ZH (2013). Survey of professional ethics observance degree among managers and staff of teaching hospitals of Shiraz University of Medical Sciences. J Adv Med Educ Prof.

[34] Faisal I, Matinnia N, Hejar A, Khodakarami Z (2014). Why do primigravidae request caesarean section in a normal pregnancy? A qualitative study in Iran. Midwifery.

[35] Farajkhoda T, Roudsari RL, Abbasi M (2012). Ethical performance in delivery of sexual and reproductive health services: a Delphi study focused on the right of confidentiality. Iran J Reprod Med.

[36] Borhani F, Alhani F, Mohammadi E, Abbaszadeh A (2010). Professional ethical competence in nursing: the role of nursing instructors. J Med Ethics Hist Med.

[37] Sheikhtaheri A, Jabali MS, Dehaghi ZH (2016). Nurses' knowledge and performance of the patients' bill of rights. Nurs ethics.

[38] Khodaparast AH, Abdolahzadeh A, Rasekh M (2008). A critical study of the “Six Ethical Codes for Research” in Iran. J Reprod Infertil.

